# Influencing factors of cognitive frailty in older patients with esophageal cancer undergoing chemotherapy: a mixed-methods study

**DOI:** 10.3389/fonc.2026.1843050

**Published:** 2026-06-16

**Authors:** Fan Wang, Zhie Gu, Yixin Jia, Yuhui Li, Quan Mao, Yusheng Shu

**Affiliations:** 1 School of Nursing, Faculty of Medicine, Yangzhou University, Yangzhou, China; 2 Department of Education and Training, Northern Jiangsu People’s Hospital, Yangzhou, China; 3 Department of Thoracic Surgery, Northern Jiangsu People’s Hospital, Yangzhou, China

**Keywords:** chemotherapy, cognitive frailty, esophageal cancer, influencing factors, mixed-methods study, older adults

## Abstract

**Objective:**

To examine the prevalence of cognitive frailty and its associated factors among older patients with esophageal cancer undergoing chemotherapy, and to provide evidence for the development of targeted nursing interventions.

**Methods:**

Based on the biopsychosocial model, an explanatory sequential mixed-methods study design was employed. A convenience sample of 246 older patients with esophageal cancer receiving chemotherapy was recruited from two tertiary general hospitals in Yangzhou City between July and December 2025. Data were collected using a general information questionnaire, the Frailty Phenotype Scale, the Montreal Cognitive Assessment, the Nutritional Risk Screening 2002 (NRS-2002), the Athens Insomnia Scale, the 15-item Geriatric Depression Scale (GDS-15), and age-adjusted Charlson comorbidity index (aCCI). Binary logistic regression analysis was performed to identify factors associated with cognitive frailty. From January to February 2026, 15 patients who met the criteria for cognitive frailty were purposively selected from the quantitative sample to participate in semi-structured interviews. Qualitative data were analyzed using Colaizzi’s seven-step method.

**Results:**

The incidence of cognitive frailty among older patients with esophageal cancer undergoing chemotherapy was 45.1%. Quantitative analysis identified chemotherapy cycles, nutritional risk, sleep disturbances, depression, educational attainment, participation in cognitive training activities, and social support as significant factors associated with cognitive frailty (*p* < 0.05).

**Conclusion:**

Cognitive frailty is relatively common among older patients undergoing chemotherapy for esophageal cancer and is influenced by multiple interrelated factors. Healthcare professionals should strengthen assessment and management during chemotherapy and develop targeted interventions to reduce the risk of cognitive frailty.

## 1 Introduction

With the rapid aging of the population in China, the number of older patients with malignant tumors has increased steadily in recent years (1). Esophageal cancer (EC) is one of the most common malignancies of the digestive tract and ranks sixth in incidence and fifth in mortality among all cancers worldwide (2). The disease predominantly affects older adults (3). Chemotherapy remains a major treatment modality for EC; however, treatment-related toxicities may aggravate functional decline and accelerate the progression of frailty (4). Evidence suggests that cancer patients undergoing chemotherapy are at increased risk of cognitive impairment (5). Frailty and cognitive impairment frequently coexist and interact, jointly influencing clinical outcomes (6). The simultaneous presence of physical frailty and mild cognitive impairment (MCI), in the absence of dementia, is generally defined as cognitive frailty (CF) (7–9). CF has been conceptualized as a transitional state between robust aging and adverse health outcomes, such as disability and dementia (10). Importantly, the conceptual framework and operational criteria of CF remain under continuous refinement and are still evolving in the current literature (11–13).

Previous studies have reported that the prevalence of CF among older patients with cancer ranges from 8.2% to 56.2% (14–18). In the general older population, CF is significantly associated with reduced activities of daily living and impaired quality of life, and is linked to an increased risk of adverse outcomes, including hospitalization, falls, dementia, and mortality (19). In contrast, patients with cancer are generally more physiologically vulnerable due to disease aggressiveness and the toxic effects of anticancer treatments, which may further exacerbate cognitive frailty-related adverse outcomes (20). Current evidence suggests that CF may be a potentially modifiable condition (21). In studies of conditions associated with CF, early identification and intervention have been associated with improved patient outcomes (22, 23), underscoring the importance of timely detection in clinical practice.

Older patients with EC receiving chemotherapy may face an even greater risk of CF due to age-related physiological decline, nutritional impairment, and cumulative treatment burden. Existing evidence indicates that advanced age, sex, educational attainment, nutritional status, and depressive symptoms are closely associated with CF and may independently contribute to its development (24, 25). However, current research has primarily focused on the general older cancer population or specific cancer types, including lung cancer and colorectal cancer (14, 16–18). Evidence regarding CF among older patients with EC undergoing chemotherapy remains limited, and the applicability of previously identified risk factors in this population is still unclear.

The biopsychosocial model proposes that the onset and progression of disease arise from the combined influence of biological, psychological, and social factors (26). As a complex clinical syndrome, CF results from multidimensional interactions, which are difficult to comprehensively reflect using a single quantitative or qualitative approach. Therefore, guided by the biopsychosocial model, this study adopts an explanatory sequential mixed-methods design to systematically examine the status of CF and its associated factors among older patients with EC undergoing chemotherapy. The findings are expected to provide evidence to support the early identification of individuals at high risk of CF and the development of targeted interventions in clinical practice.

## 2 Material and method

### 2.1 Population and sample of the study

#### 2.1.1 Quantitative phase

Convenience sampling was used to recruit older patients with EC who were admitted to the thoracic surgery and oncology wards of two tertiary Grade A general hospitals (the highest level of hospital accreditation in China) in Yangzhou, Jiangsu Province, China, between July and December 2025. The study population encompassed patients across different clinical stages of EC.

The inclusion criteria were as follows: (1) pathologically confirmed primary esophageal cancer; (2) age ≥60 years, which is commonly used in geriatric and epidemiological studies in China; (3) completion of at least one cycle of chemotherapy; and (4) awareness of the diagnosis and provision of written informed consent.

The exclusion criteria included: (1) esophageal cancer representing metastases from other primary sites; (2) presence of brain metastases; (3) severe hearing or communication impairment; (4) coexisting psychiatric disorders; (5) history of dementia; (6) terminal-stage cancer receiving palliative care; (7) current use of medications known to significantly affect cognitive function, including long-term or regular use of benzodiazepines, anticholinergic drugs, tricyclic antidepressants, antipsychotics, antiepileptic drugs, and opioid analgesics.

According to sample size estimation principles, the required sample size is generally 10–20 times the number of independent variables included in the analysis (27). A total of 17 independent variables were considered in this study. After accounting for an anticipated attrition rate of approximately 10%, the estimated sample size ranged from 189 to 378 participants.

#### 2.1.2 Qualitative phase

Between January and February 2026, older patients with EC undergoing chemotherapy who met the criteria for CF were purposively selected from the quantitative study sample. Semi-structured interviews were conducted using a maximum variation sampling strategy, with consideration given to differences in sex, age, and educational attainment. The inclusion and exclusion criteria were consistent with those applied in the quantitative phase. Data collection continued until thematic saturation was achieved, defined as the point at which no new themes or meaningful information emerged from subsequent interviews (28).

### 2.2 Research tools

#### 2.2.1 Quantitative phase

##### 2.2.1.1 Assessment of independent variables

(1) Demographic and clinical characteristics questionnaire (self-developed).

The questionnaire was self-developed by the research team based on a review of the literature. It collected data on patient age, body mass index (BMI), marital status, educational attainment, dietary habits, polypharmacy status, disease stage, number of chemotherapy cycles, and clinical indicators, including hemoglobin, albumin, and platelet counts.

(2) Geriatric depression scale-15 (GDS-15).

The scale was simplified by Schneider et al. (29) comprises 15 items grouped into four domains: sadness, apathy and anxiety, loss of hope, and memory impairment with reduced social activity. Ten items are scored using a “yes/no” format, with each item assigned 0 or 1 point, while the remaining five items are reverse-scored. A total score of ≥ 5 indicates the presence of depressive symptoms, with higher scores reflecting greater severity. The Chinese version demonstrates good internal consistency, with a Cronbach’s α of 0.793 (30).

(3) Athens insomnia scale (AIS).

The Athens Insomnia Scale (AIS), developed by Soldatos et al. (31), was translated into Chinese by Sun et al. (32). The scale assesses sleep status over the past month and consists of eight items covering sleep induction, nocturnal awakenings, early morning awakening, total sleep duration, overall sleep quality, well-being, daytime functioning, and daytime sleepiness. Each item is rated on a 4-point Likert scale ranging from 0 to 3, yielding a total score between 0 and 24. Scores of < 4 indicate no sleep disturbance, 4–6 suggest possible sleep disturbance, and ≥ 7 indicate insomnia. The Chinese version demonstrates good internal consistency, with a Cronbach’s α of 0.83 (32).

(4) Nutritional risk screening 2002 (NRS-2002).

The Nutritional Risk Screening 2002 (NRS-2002), developed by Kondrup et al. (33) and recommended by the European Society for Clinical Nutrition and Metabolism (ESPEN), was used to assess nutritional risk. The scale consists of three components: impaired nutritional status, disease severity, and age adjustment. A total score of < 3 indicates no nutritional risk, whereas a score of ≥ 3 indicates the presence of nutritional risk. The instrument has demonstrated good reliability in clinical nutritional screening, with a Cohen’s k of 0.72 (34).

(5) Age-adjusted Charlson comorbidity index (aCCI).

The age-adjusted Charlson Comorbidity Index (aCCI), first proposed by Charlson et al. in 1994 (35), is a widely adopted tool for quantifying comorbidity burden in clinical settings. It assigns weighted scores to chronic medical conditions based on disease type and severity, with individual comorbidities rated at 1, 2, 3, or 6 points respectively. Meanwhile, age is divided into six strata: < 50, 50–< 60, 60–< 70, 70–< 80, 80–< 90, and ≥ 90 years, which are assigned 0, 1, 2, 3, 4, and 5 points in sequence. The total aCCI score represents the overall comorbidity burden, whereby higher scores indicate a more severe comorbidity condition.

##### 2.2.1.2 Assessment of CF

(1) Frailty phenotype (FP).

The Frailty Phenotype (FP), developed by Fried et al. (36), was used to assess frailty status. The scale includes five components: unintentional weight loss, slow walking speed, weak grip strength, low physical activity, and self-reported exhaustion. Each criterion met is assigned 1 point, resulting in a total score ranging from 0 to 5. Scores of 0 indicate robustness, 1–2 indicate pre-frailty, and ≥ 3 indicate frailty. The scale has demonstrated good reliability and validity, with a Cronbach’s α of 0.821 (37).

(2) Montreal cognitive assessment (MoCA).

The Montreal Cognitive Assessment (MoCA), developed by Nasreddine et al. (38), was used to assess cognitive function. The Chinese Beijing version (MoCA-BJ), translated and culturally adapted by Yang et al. (39), was adopted in this study. The scale includes eight cognitive domains: visuospatial and executive function, naming, memory, attention, language, abstraction, delayed recall, and orientation, with a maximum total score of 30. A score of < 26 indicates cognitive impairment. One additional point is added for individuals with ≤ 12 years of education, with the total score capped at 30. The Chinese version demonstrates good internal consistency, with a Cronbach’s α of 0.81 (39).

Based on the Chinese classification of the Montreal Cognitive Assessment proposed by Zhang et al. (40), CF was defined as the coexistence of physical frailty (Frailty Phenotype score ≥ 3) and mild cognitive frailty (MoCA score of 15–24) in the absence of dementia.

#### 2.2.2 Qualitative phase

Based on the findings of the quantitative phase, an initial interview guide was developed by the research team in accordance with the biopsychosocial medical model. Preliminary interviews were conducted with three EC patients who met the inclusion criteria to assess the clarity and feasibility of the interview questions. The guide was subsequently revised and refined based on feedback from these interviews and recommendations from clinical physicians, resulting in the final interview guide. The main interview questions included:

1. During chemotherapy, have you experienced any changes in your physical or cognitive function? If so, could you describe these changes?
2. What factors do you believe contributed to these changes? How did you cope with them? Are you aware of any strategies to prevent or delay such changes?

### 2.3 Data collection and quality control

#### 2.3.1 Questionnaire administration and quality control

Prior to data collection, all investigators received standardized training regarding the study objectives, research procedures, scale scoring criteria, and the standardized use of dynamometers. Eligible hospitalized patients with EC were recruited according to the predefined inclusion and exclusion criteria. Questionnaires were administered through face-to-face, one-on-one interviews. Standardized instructions were provided to explain the study purpose and procedures, and questionnaires were distributed after written informed consent was obtained. All questionnaires were completed anonymously.

For participants unable to complete the questionnaire independently due to advanced age, limited educational attainment, or reading difficulties, trained investigators provided assistance after standardized explanations while avoiding any leading guidance. Completed paper-based questionnaires were collected immediately and checked for completeness and accuracy, with missing or inconsistent responses verified on site. Clinical data were extracted from medical records and recorded uniformly by the researchers.

#### 2.3.2 Interview procedure and quality control

Face-to-face semi-structured interviews were conducted by two graduate nursing students who had received formal training in qualitative research methods and possessed prior interview experience. Interviews were carried out in quiet and private settings, such as demonstration classrooms or ward activity rooms. Before the interview, researchers introduced themselves, explained the study purpose and procedures, informed participants that the interview would be audio-recorded, and obtained written informed consent.

To ensure confidentiality, all interview data were anonymized using coded identifiers. During the interview, one researcher served as the primary interviewer, asking flexible questions based on the interview guide and participants’ responses while avoiding leading prompts. The second researcher was responsible for observing and documenting nonverbal cues, including facial expressions, emotional responses, and body language. Each interview lasted approximately 30–40 minutes. Audio recordings were transcribed verbatim within 24 hours after the interview, and unclear information was verified with participants when necessary.

### 2.4 Statistical methods

#### 2.4.1 Quantitative research

Statistical analyses were performed using IBM SPSS Statistics version 29.0. Categorical variables were described using frequencies and percentages. Continuous variables were tested for normality using the Shapiro-Wilk test. Variables with normal distribution were expressed as mean ± standard deviation (SD), whereas non-normally distributed variables were presented as median and interquartile range (IQR). In univariate analysis, continuous variables were compared using independent samples t-tests or Mann–Whitney U tests according to data distribution, while categorical variables were analyzed using chi-square tests or Fisher’s exact tests. Variables with *p* < 0.05 in univariate analysis were entered into a multivariate binary logistic regression model to identify independent influencing factors. A two-tailed *p value <0.05* was considered statistically significant.

#### 2.4.2 Qualitative research

Interview data were organized and managed using NVivo 20 software. Data analysis was conducted following the seven-step phenomenological method proposed by Colaizzi (41). First, interview transcripts were read repeatedly to obtain an overall understanding of participants’ experiences. Significant statements related to CF were extracted and coded. Similar meanings were formulated and clustered into themes, followed by exhaustive descriptions of the phenomenon. The resulting thematic structure was further refined and validated by returning to participants for verification to ensure credibility. All interview data were independently coded and analyzed by at least two researchers. Any discrepancies were resolved through research team discussions to ensure analytical rigor and consistency.

#### 2.4.3 Integration of mixed-methods data

An explanatory sequential mixed-methods design was employed in this study. Quantitative analysis was conducted initially to identify factors associated with CF among older patients with EC undergoing chemotherapy. Subsequently, qualitative interviews were performed to further interpret and enrich the quantitative findings. Quantitative and qualitative data were then integrated using a joint display approach to examine areas of convergence, complementarity, and divergence between statistical results and qualitative themes related to factors influencing CF. This integrative process facilitated the development of comprehensive inferences and strengthened the overall interpretation of the findings.

### 2.5 Ethics

This study was approved by the Ethics Committee of Yangzhou University (approval number: YXYLL-2025-137) and conducted in accordance with the principles of the Declaration of Helsinki. All participants provided written informed consent and were informed of their right to withdraw from the study at any time without any consequences.

## 3 Results

### 3.1 Quantitative research findings

#### 3.1.1 General characteristics of older EC patients

A total of 255 questionnaires were collected in this study. Among them, 9 were excluded due to incomplete scale responses, missing key variables, or withdrawal of participants during the survey, which prevented complete assessment of the primary outcome measures. Finally, 246 valid questionnaires were included, yielding a valid response rate of 96.5%.

Among the 246 older patients with EC, 193 were male (78.5%) and 53 were female (21.5%). Most patients had squamous cell carcinoma (n = 239, 97.2%), while 7 (2.8%) had adenocarcinoma. In terms of anticancer treatment, the majority received platinum plus taxane-based chemotherapy regimens. Some patients received additional immunotherapy, radiotherapy, or targeted therapy. Commonly used chemotherapeutic agents included cisplatin, nedaplatin, lobaplatin, and albumin-bound paclitaxel, while immunotherapy agents mainly included tislelizumab and camrelizumab. Overall, treatment patterns were mainly based on combination regimens, including chemotherapy combined with immunotherapy, chemotherapy combined with radiotherapy and immunotherapy, and postoperative combination therapy.

The results showed that 111 patients (45.1%) developed CF. Assessment using the Frailty Phenotype scale indicated that 86 patients were non-frail, whereas 160 patients were classified as frail, yielding a frailty prevalence of 65.0%. MoCA results showed that 46 patients scored ≥ 25, while 200 patients scored < 25, indicating that most older patients with EC experienced varying degrees of cognitive decline during chemotherapy. Among the 135 patients without CF, 36 were non-frail with MoCA ≥ 25, 50 were non-frail with MoCA < 25; among those with frailty, 10 had MoCA ≥ 25 and 39 had MoCA < 25. The remaining demographic and clinical characteristics are presented in **Table 1**.

**Table 1:** General characteristics and univariate analysis of factors associated with CF in older patients with EC undergoing chemotherapy (n = 246).

Variables		CF group (n=111)		Non-CF group (n=135)		Statistic	*P*
		n	%	n	%		
Age	60-70	52	(46.8)	66	(48.9)	0.379^1^	0.84
	71-80	56	(50.5)	64	(47.4)		
	81-90	3	(2.7)	5	(3.7)		
Gender	Man	94	(84.7)	99	(73.3)	4.644^1^	0.031
	Woman	17	(15.3)	36	(26.7)		
Marital status	Married	90	(81.1)	114	(84.4)	0.487^1^	0.485
	Unmarried/Divorced/Widowed	21	(18.9)	21	(15.6)		
Educational attainment	Primary school or below	60	(54.1)	85	(63)	9.422^1^	0.024
	Junior high school	34	(30.6)	34	(25.2)		
	Senior high school or technical secondary school	15	(13.5)	7	(5.2)		
	College degree or above	2	(1.8)	9	(6.7)		
Polypharmacy	< 5	108	(97.3)	131	(97)	0.000^1^	1.000
	≥ 5	3	(2.7)	4	(3)		
Tumor stage	I	2	(1.8)	3	(2.2)	0.522^1^	0.935
	II	14	(12.6)	15	(11.1)		
	III	35	(31.5)	47	(34.8)		
	IV	60	(54.1)	70	(51.9)		
Chemotherapy cycles	< 3 cycles	40	(36)	75	(55.6)	9.323^1^	0.002
	≥ 3 cycles	71	(64)	60	(44.4)		
Dietary habits	Normal diet	67	(60.4)	96	(71.1)	3.149^1^	0.076
	Unhealthy diet	44	(39.6)	39	(28.9)		
Charlson comorbidity index	Low risk	0	(0)	0	(0)	0.664^1^	0.717
	Moderate risk	38	(34.2)	50	(37.0)		
	High risk	57	(51.4)	70	(51.9)		
	Severe risk	16	(14.4)	15	(11.1)		
Nutritional risk	No	47	(42.3)	75	(55.6)	4.254^1^	0.039
	Yes	64	(57.7)	60	(44.4)		
Sleep disturbance	No	13	(11.7)	34	(25.2)	7.155^1^	0.007
	Yes	98	(88.3)	101	(74.8)		
Depression	No	15	(13.5)	43	(31.9)	11.369^1^	<0.001
	Yes	96	(86.5)	92	(68.1)		
Participation in cognitive training activities	No	76	(68.5)	48	(35.6)	26.396^1^	<0.001
	Yes	35	(31.5)	87	(64.4)		
BMI (kg/m²)		20.96 ± 2.75		20.68 ± 3.19		-0.739^2^	0.461
Hemoglobin (g/L)		111.76 ± 18.93		114.07 ± 19.71		0.931^2^	0.353
Albumin (g/L)		40.4(37.35,42.5)		41.4(38.5,44.0)		-2.118^3^	0.034
Platelet count (×10^9^/L)		149(114.5,202.5)		175(129.5,225.5)		-2.552^3^	0.011

¹Chi-square test. When the assumptions of the chi-square test were not met, continuity correction chi-square test or Fisher’s exact test was applied. For larger tables with multiple rows and columns, the Fisher–Freeman–Halton exact test was used.

²Independent samples t-test.

³Mann–Whitney U test.

#### 3.1.2 Univariate analysis of factors influencing cognitive function in older EC patients

Univariate analysis indicated that gender, educational attainment, number of chemotherapy cycles, nutritional risk, sleep disturbance, depression, participation in cognitive training activities, albumin (g/L), and platelet count (×10^9^/L) were significantly associated with CF in older patients with EC undergoing chemotherapy (*p* < 0.05). Detailed results are summarized in **Table 1**.

#### 3.1.3 Multicollinearity diagnosis

Multicollinearity was assessed for the nine variables that were statistically significant in the univariate analysis. The results indicated no significant multicollinearity among these variables, allowing their inclusion in the subsequent binary logistic regression analysis. Detailed results of the multicollinearity assessment are presented in **Table 2**.

**Table 2:** Multicollinearity analysis of independent variables.

Variables	TOL	VIF
Gender	0.848	1.18
Educational attainment	0.87	1.15
Chemotherapy cycles	0.864	1.157
Albumin (g/L)	0.853	1.172
Platelet count (×10^9^/L)	0.913	1.096
Nutritional risk	0.868	1.152
Sleep disturbance	0.899	1.112
Depression	0.886	1.129
Participation in cognitive training activities	0.908	1.102

All variables showed tolerance (TOL) values > 0.1 and variance inflation factor (VIF) values < 10, indicating that no significant multicollinearity was detected.

#### 3.1.4 Multivariate analysis of factors influencing CF in older EC patients undergoing chemotherapy

A binary logistic regression model was established with the occurrence of CF (no = 0, yes = 1) as the dependent variable and the nine variables that were statistically significant in the univariate analysis entered as independent variables. The lowest category of each independent variable was used as the reference group, and variable assignments are presented in **Table 3**.

**Table 3:** Assignment of categorical independent variables.

Variables	Assignment method
Age	60–70 = 1; 70–80 = 2; 80–90 = 3
Gender	Man = 1; Woman = 2
Marital status	Married = 1; Unmarried/Divorced/Widowed = 2
Educational attainment	Primary school or below = 1; Junior high school = 2; Senior high school or technical secondary school = 3; College degree or above = 4
Polypharmacy	< 5 = 1; ≥ 5 = 2
Tumor stage	Stage I = 1; Stage II = 2; Stage III = 3; Stage IV = 4
Chemotherapy cycles	< 3 cycles = 1; ≥ 3 cycles = 2
Dietary habits	Normal diet = 1; Unhealthy diet = 2
Charlson comorbidity index	Low risk = 1; Moderate risk = 2; High risk = 3; Severe risk = 4
Nutritional risk	No = 0; Yes = 1
Sleep disturbance	No = 0; Yes = 1
Depression	No = 0; Yes = 1
Participation in cognitive training activities	No = 0; Yes = 1

The results demonstrated that educational attainment (college degree or above, OR = 0.108, 95% CI: 0.016–0.725), chemotherapy cycles (≥ 3 cycles, OR = 2.421, 95% CI: 1.257–4.662), nutritional risk (yes, OR = 2.201, 95% CI: 1.152–4.207), sleep disturbance (yes, OR = 2.536, 95% CI: 1.093–5.889), depression (yes, OR = 2.958, 95% CI: 1.351–6.475), and participation in cognitive training activities (yes, OR = 0.140, 95% CI: 0.070–0.282) were independently associated with CF in older EC patients undergoing chemotherapy.

All associations were statistically significant (*p* < 0.05), as shown in **Table 4**.

**Table 4:** Multivariate analysis of factors associated with CF in older patients with EC undergoing chemotherapy.

Variables	B	SE	Waldχ^2^	*P*	Exp (B)	95%CI
Constant	-1.029	1.666	0.381	0.537	0.357	–
Educational attainment (College degree or above)	-2.23	0.973	5.246	0.022	0.108	0.016-0.725
Chemotherapy cycles (≥ 3 cycles)	0.884	0.334	6.993	0.008	2.421	1.257-4.662
Nutritional risk (yes)	0.789	0.33	5.701	0.017	2.201	1.152-4.207
Sleep disturbance (yes)	0.931	0.43	4.691	0.03	2.536	1.093-5.889
Depression (yes)	1.084	0.4	7.357	0.007	2.958	1.351-6.475
Participation in cognitive training activities (yes)	-1.964	0.357	30.312	<0.001	0.14	0.07-0.282

B, regression coefficient; SE, standard error; Waldχ2, Wald chi-square statistic; *p*, p-value; OR, odds ratio; 95% CI, 95% confidence interval.

### 3.2 Qualitative research findings

A total of 15 participants were included in the qualitative study. The age distribution comprised 7 participants aged 60–70 years, 7 aged 70–80 years, and 1 aged 81–90 years. The sample included 8 males and 7 females. Regarding educational attainment, 7 participants had primary school education or below, 4 had junior high school education, 2 had senior high school or technical secondary education, and 2 had college education or above. In terms of chemotherapy exposure, 6 participants had undergone fewer than 3 chemotherapy cycles, while 9 had completed 3 or more cycles.

#### 3.2.1 Dual impact of treatment-related physical burden on physical and cognitive function

##### 3.2.1.1 Prolonged chemotherapy exacerbates dual physical and cognitive impairment

Most participants reported that with increasing chemotherapy cycles, treatment-related adverse effects gradually accumulated, leading to reduced physical endurance accompanied by perceived cognitive decline, particularly difficulties in concentration and slowed thinking.

P1: “The first week after each chemo cycle brings extreme fatigue requiring prolonged bed rest. I feel chilly and my overall condition is poor—my mind feels foggy too.”

P7: “After chemo, my body and memory feel weaker than before. I tire easily after brief activity, and my reactions have slowed considerably.”

Several participants further reported aggravated physical discomfort following chemotherapy and local treatments, with persistent eating difficulties emerging as an ongoing concern. Decreased appetite and noticeable weight loss were commonly accompanied by reduced physical strength and self-perceived cognitive decline.

P8: “During chemotherapy and radiotherapy, eating became difficult. My appetite decreased significantly, and I lost over 5 kg. I barely had the strength to walk and felt completely exhausted.”

P10: “I often worry while eating, fearing blockage or reflux. My food intake has decreased noticeably, leaving me increasingly weak. My thinking and reactions are slower than before.”

##### 3.2.1.2 Sleep impairment aggravates fatigue and cognitive decline

In addition to treatment-related physical discomfort, many participants reported substantial sleep disturbances during chemotherapy. Common manifestations included difficulty initiating sleep, frequent nocturnal awakenings, and disrupted sleep patterns.

Persistent sleep problems were associated with sustained daytime fatigue, reduced activity levels, and perceived declines in attention, memory, and responsiveness.

P5: “After treatment, my sleep rhythm was severely disrupted. It was hard to fall asleep, and sleep was very light. During the day, I felt mentally sluggish, with noticeably slower brain responses and poorer memory.”

P6: “Nightly awakenings from coughing and nausea repeatedly interrupt sleep, leaving me exhausted the next day with poor concentration and slowed reactions.”

Some participants indicated that postoperative positioning requirements and gastroesophageal reflux further interfered with nighttime rest, contributing to ongoing physical exhaustion and reduced mental vitality.

P14: “Post-surgery, prolonged semi-recumbent rest is required, yet frequent acid reflux persists, causing poor sleep at night and a clear decline in both physical condition and mental state.”

#### 3.2.2 Psychological coping processes and perceived changes in cognitive function

##### 3.2.2.1 The cognitive impact of illness uncertainty and negative emotions

Several participants described experiencing anxiety, fear, uncertainty, and helplessness during treatment. Limited understanding of the disease trajectory, treatment procedures, and prognosis contributed to persistent psychological distress. Many reported remaining in a prolonged state of tension, which they perceived as affecting concentration, mental clarity, and memory.

P9: “When I was diagnosed, I had difficulty accepting it psychologically. I couldn’t sleep well for a long time, my emotions fluctuated, and my overall condition worsened.”

P12: “After chemotherapy, my psychological burden increased. I constantly paid attention to treatment progress and physical recovery. It affected my sleep and appetite, and I felt my memory wasn’t as good as before.”

Participants also described heightened anxiety while awaiting examination results or treatment decisions. The uncertainty associated with repeated testing was perceived as mentally exhausting and disruptive to daily functioning.

P3: “Before each chemotherapy session, I had to wait for test results. That waiting period was extremely stressful. I could hardly sleep and kept worrying that abnormal results would delay the next treatment.”

P11: “My mood stayed low for a long time. Every day felt difficult. I found it hard to concentrate and clearly felt that my thinking had slowed down.”

##### 3.2.2.2 Positive regulation through psychological adjustment and disease acceptance

As treatment progressed, some participants described gradually adjusting their mindset. Acceptance of the illness and renewed confidence in treatment were perceived as helping them stabilize their emotions and reduce psychological strain.

Participants with higher educational attainment noted that actively seeking disease-related information improved their understanding of treatment and enhanced their sense of control. This was accompanied by self-reported improvements in emotional stability and mental clarity.

P5: “I actively watch medical science videos to learn about the latest treatments. Medical technology is advancing quickly, and treatment outcomes are improving. That gives me more confidence.”

P8: “I think mindset matters a lot.At first, I felt very depressed after the diagnosis. But gradually I accepted it and adjusted my attitude. After cooperating actively with treatment, both my physical and mental state improved compared with before.”

#### 3.2.3 Social support and daily activity participation in relation to CF

##### 3.2.3.1 Social interaction and enriching activities maintain cognitive vitality

Participants frequently described maintaining involvement in daily activities such as reading, watching the news, chatting with others, playing chess, or participating in community events. These activities were perceived as helping them stay mentally active. Some participants noted that prolonged periods at home or reduced social interaction were accompanied by feelings of slowed thinking and forgetfulness. In contrast, regular engagement in social and mentally stimulating activities was described as helping them maintain mental clarity.

P6: “I regularly join community activities like playing the erhu or card games. If I stay at home for too long, my thinking feels dull. These activities keep my mind active.”

P7: “I enjoy reading, walking, and playing chess. These activities help me keep my thinking clear and respond more quickly.”

##### 3.2.3.2 Family and healthcare support enhance security and preserve cognitive function

Participants emphasized the importance of family companionship and support from healthcare professionals during treatment. Family members were described as providing practical assistance and emotional reassurance, while medical staff were seen as offering professional guidance and timely communication. Such support was perceived as helping patients feel more secure and emotionally stable during treatment.

P2: “Without my spouse taking care of my daily needs, I wouldn’t be able to manage many things.”

P1: “When I feel uncomfortable during chemotherapy or radiotherapy, I communicate with my doctor right away. Their explanations make me feel much more at ease.”

### 3.3 Mixed-methods integration

The findings from the quantitative and qualitative phases were integrated, and the combined results are presented in **Table 5**.

**Table 5:** Integration of quantitative and qualitative findings.

Theoretical model	Quantitative findings	Qualitative findings	Integrated results
Biological	≥ 3 chemotherapy cycles significantly increased the risk of CF (β = 0.789, p = 0.017).	The theme “Prolonged chemotherapy exacerbates dual physical and cognitive impairment” reflected the cumulative toxic effects of chemotherapy over time, leading to reduced physical tolerance accompanied by decreased attention and mental clarity.	Convergence
Biological	Nutritional risk significantly increased the risk of CF (β = 0.789, *p* = 0.017).	The same theme highlighted restricted food intake, decreased appetite, and significant weight loss during chemoradiotherapy, resulting in physical decline, limited daily activities, and subjective cognitive inefficiency.	Expansion
Biological	Sleep disturbance significantly increased the risk of CF (β = 0.931, *p* = 0.030).	The theme “Sleep impairment aggravates fatigue and cognitive decline” described difficulty initiating sleep, nocturnal awakening, and circadian rhythm disruption, leading to persistent fatigue and impaired attention, reaction speed, and memory.	Convergence
Psychological	Depression significantly increased the risk of CF (β = 1.084, *p* = 0.007).	The theme “The cognitive impact of illness uncertainty and negative emotions” indicated that insufficient disease knowledge and sustained negative emotions placed patients in prolonged psychological distress, resulting in distractibility and reduced cognitive efficiency.	Convergence
Social	Higher educational attainment (college degree or above) was a protective factor for CF (β = -2.23, *p* = 0.022).	The theme “Positive regulation through psychological adjustment and disease acceptance” reflected that patients with higher education levels were more likely to actively seek disease-related information, adjust coping strategies, and maintain cognitive engagement.	Expansion
Social	Participation in cognitive training activities was a protective factor for CF (β = –1.964, *p* < 0.001).	The theme “Social interaction and enriching activities maintain cognitive vitality” suggested that sustained cognitive stimulation helps preserve mental agility.	Convergence
Social	——	The theme “Family and healthcare support enhance security and preserve cognitive function” supplemented the quantitative model by highlighting patients’ experiences of social support, suggesting its potential protective role in the development of CF.	Expansion

## 4 Discussion

The present study identified a 45.1% incidence of CF among older EC patients undergoing chemotherapy, which exceeds the prevalence reported by Wang Hui et al. (14) in the general older cancer population. This finding indicates that this subgroup may be at particularly high risk of CF. The relatively high prevalence of frailty and cognitive impairment observed in this study may be partly attributed to the generally low educational attainment of the participants, including a proportion who had received no formal education. In addition, disease-related factors such as impaired dietary intake, malnutrition, and cumulative chemotherapy-related toxicity may also contribute to the development of frailty and cognitive decline in patients with EC (42).

Qualitative findings further deepened this understanding by highlighting patients’ lived experiences of chemotherapy-related physical burden, psychological coping processes, and social support dynamics. The integration of quantitative and qualitative data demonstrated convergence in the identification of key influencing factors, while qualitative insights expanded upon quantitative associations by clarifying underlying mechanisms and contextualizing individual variability. Together, these findings provide a more comprehensive and nuanced understanding of CF within this specific clinical context.

Previous evidence, mainly derived from non-oncologic or general older adult populations, suggests that early identification and timely intervention for CF can substantially reduce adverse health outcomes in older adults (43–45). Given that older patients may have limited awareness of functional decline due to advanced age, lower educational attainment, and restricted access to health information, clinical practice should prioritize the implementation of individualized and comprehensive cognitive assessment strategies. Additionally, targeted health education interventions are warranted to enhance health literacy and strengthen patients’ self-management capacities.

### 4.1 Physiological factors: extended chemotherapy cycles, nutritional risk, and sleep disturbance

The present study identified prolonged chemotherapy exposure as an independent risk factor for CF in older EC patients, with risk increasing alongside the number of treatment cycles. This finding is consistent with both our qualitative data and previous research (18). In China, EC is predominantly squamous cell carcinoma, and platinum-based chemotherapy remains the standard first-line regimen (46). Platinum agents are known to exert neurotoxic effects by increasing oxidative stress, inducing DNA damage, and disrupting mitochondrial function, thereby contributing to cognitive decline and cancer-related cognitive impairment (47). Longitudinal evidence further suggests that cognitive function progressively deteriorates following chemotherapy initiation, and the likelihood of impairment rises with cumulative exposure (48, 49). These findings indicate that repeated chemotherapy may act as a sustained biological stressor, accelerating the simultaneous progression of physical frailty and cognitive vulnerability. Therefore, comprehensive baseline cognitive assessments should be implemented prior to treatment initiation to identify high-risk individuals and enable early intervention. Moderate-intensity physical activity (e.g., Tai Chi, Baduanjin) and structured cognitive training programs have been shown to be beneficial in non-oncologic older adult populations, and may help mitigate treatment-related functional decline (45, 50).

Nutritional risk also emerged as a significant contributor to CF, corroborating the findings of Wang et al. (25). Patients with compromised nutritional status exhibited a markedly elevated CF risk, and qualitative narratives further illustrated how reduced appetite, restricted intake, and weight loss were accompanied by diminished physical endurance and subjective cognitive inefficiency. Older patients undergoing chemotherapy are particularly vulnerable to sustained energy deficits due to dysphagia, treatment-related gastrointestinal toxicity, and cancer-associated metabolic alterations (51). Malnutrition may reduce physiological reserve, aggravate systemic inflammation, and impair neuronal function, thereby linking physical frailty with cognitive deterioration. Evidence supports a strong association between inadequate protein and micronutrient intake and cognitive decline (24). Accordingly, routine nutritional screening and dynamic monitoring should be integral components of chemotherapy care. Nutritional strategies emphasizing adequate energy and protein intake, alongside dietary patterns rich in unsaturated fatty acids and antioxidants (e.g., Mediterranean-style diet), may be beneficial (52). For individuals with severe dysphagia or insufficient oral intake, timely enteral nutritional support should be considered to minimize CF risk (23).

Sleep disturbance represented another critical physiological correlate of CF, with quantitative and qualitative findings demonstrating consistent associations, in agreement with prior literature (53, 54). Chronic insomnia and sleep fragmentation may contribute to CF through neuroinflammatory activation, impaired glymphatic clearance of neurotoxic metabolites, and dysregulation of circadian rhythms (55). Conversely, cognitive decline may further disrupt sleep architecture, creating a bidirectional cycle that perpetuates functional deterioration. These findings underscore the importance of systematic sleep assessment and ongoing monitoring during chemotherapy. Non-pharmacological interventions, including sleep hygiene education, optimization of sleep environment, and structured sleep scheduling, should be prioritized to improve sleep quality and potentially attenuate CF progression (54).

### 4.2 Psychological factors: depression

The present study identified depression as a significant psychological determinant of CF in older EC patients, consistent with previous findings (17). Quantitative analysis demonstrated that depressive symptoms were associated with a markedly increased risk of CF. Qualitative interviews further illustrated that illness-related uncertainty and persistent negative emotions intensified psychological stress, impaired attentional control, and reduced memory efficiency, thereby accelerating cognitive vulnerability.

EC is often insidious in onset and highly invasive, with many patients diagnosed at intermediate or advanced stages (56, 57). Concerns regarding disease progression, treatment burden, and prognosis may readily trigger depressive symptoms (58). From a mechanistic perspective, depression may contribute to cognitive decline through multiple pathways, including chronic inflammatory activation, dysregulation of the hypothalamic–pituitary–adrenal axis, and increased oxidative stress, all of which can adversely affect neuronal function and neuroplasticity (59). Moreover, depressive states may diminish motivation, reduce engagement in daily and social activities, and impair treatment adherence, thereby further limiting cognitive stimulation and functional reserve (60).

Accordingly, early screening and dynamic monitoring of depressive symptoms should be incorporated into routine oncology nursing care. Non-pharmacological psychological interventions tailored to patients’ emotional status and cognitive capacity, including music therapy (61), mindfulness-based interventions, and narrative care, may alleviate emotional distress and promote cognitive resilience. In addition, timely referral to mental health professionals for comprehensive assessment and management should be encouraged to optimize psychological well-being and potentially mitigate CF progression.

### 4.3 Social factors: educational attainment, cognitive activities, and social support

This study demonstrated that college-level education or above and active engagement in cognitively stimulating activities were associated with a reduced risk of CF among older patients with EC, consistent with previous findings (18, 62). Qualitative results further enriched these findings, indicating that patients with higher educational attainment were more inclined to proactively seek disease-related information, adjust cognitive strategies, and participate in cognitive activities to maintain cognitive function. Higher educational levels may delay neuronal aging and cognitive decline by enhancing cognitive reserve and improving synaptic transmission efficiency (63). In addition, better health literacy and stronger treatment adherence may encourage patients to actively engage in self-health management and sustained cognitive stimulation (14).

Cognitively training activities may improve cognitive performance by supporting hippocampal neuronal survival, increasing cerebral blood flow, and promoting angiogenesis (64). Beyond biological mechanisms, the cognitive challenges and social interactions embedded in these activities may help preserve psychological and social functioning, thereby delaying the onset of CF (65). Accordingly, healthcare professionals should provide stratified health education tailored to patients’ educational backgrounds to enhance treatment adherence and encourage long-term participation in appropriate cognitive and social activities, with the aim of maintaining cognitive vitality and reducing CF risk.

Qualitative findings also revealed that family companionship and timely communication from healthcare providers enhanced patients’ sense of security and confidence in treatment. These findings complement the quantitative results from the perspective of social support, suggesting that a robust support system plays a vital role in CF prevention and management, consistent with prior studies (66, 67). Positive social support may alleviate psychological stress, strengthen a sense of belonging, and promote cognitive functioning by stimulating brain activity and facilitating the release of neurotransmitters such as oxytocin and dopamine, thereby delaying CF progression (68, 69). In addition, with the increasing number of older adults living alone or without a spouse, social support should not be limited to family-based assistance but should encompass multiple sources, including healthcare professional support, community-based services, psychological counseling, and peer support from other patients (70). Therefore, nursing staff should encourage family members to provide emotional support and companionship, while also assisting patients in accessing and integrating these diverse social support resources to reduce the risk of CF.

## 5 Conclusions

This study demonstrated a relatively high prevalence of CF among older patients with EC undergoing chemotherapy. CF was significantly associated with chemotherapy cycles, nutritional risk, sleep disturbances, depression, educational attainment, participation in cognitive training activities, and social support. These findings highlight the multifactorial nature of CF within a biopsychosocial framework and underscore the importance of early identification and comprehensive risk assessment in multidisciplinary clinical practice.

### 5.1 Strengths

This study has several strengths. First, it focuses on older patients with EC undergoing chemotherapy, a clinically relevant population that remains relatively underexplored, thereby providing important complementary evidence. Second, a mixed-methods design was adopted, integrating quantitative and qualitative approaches. Guided by the biopsychosocial framework, the study systematically examined factors associated with CF from multiple dimensions, thereby enhancing the comprehensiveness and interpretability of the findings. Finally, the findings provide a basis for future research on early screening, risk stratification, and targeted interventions for CF in older patients with EC.

### 5.2 Limitations

This study has several limitations. First, its cross-sectional design precludes inference on temporal changes in CF during chemotherapy. Second, the age threshold of ≥ 60 years, although commonly used in China, differs from the ≥ 65 years definition adopted in some international studies, which may limit comparability. Third, incomplete medical records prevented systematic collection of detailed treatment-related information, including treatment lines and severe adverse events, thereby limiting further analysis of treatment-specific effects. Finally, the relatively small sample size and recruitment from two hospitals may restrict the generalizability of the findings. Future studies should adopt longitudinal, multicentre, and larger-sample designs to refine risk prediction and intervention strategies.

## Data Availability

The datasets presented in this article are not readily available because the datasets generated and/or analyzed during this study are not publicly available due to ethical and privacy restrictions but may be made available from the corresponding author upon reasonable request and with permission from the relevant ethics committee. Requests to access the datasets should be directed to Fan Wang, wfwyyy96@163.com.
